# Clinic Nonattendance Is a Risk Factor for Poor Kidney Transplant Outcomes

**DOI:** 10.1097/TXD.0000000000000836

**Published:** 2018-10-24

**Authors:** Cathy Richardson, Aimee Williams, Jill McCready, Khalid Khalil, Felicity Evison, Adnan Sharif

**Affiliations:** 1 Department of Nephrology and Transplantation, Queen Elizabeth Hospital, Edgbaston, Birmingham, United Kingdom.; 2 University of Birmingham, Birmingham, United Kingdom.; 3 Department of Health Informatics, Queen Elizabeth Hospital, Edgbaston, Birmingham, United Kingdom.

## Abstract

**Background:**

The aim of this study was to analyze the impact of clinic nonattendance within the first year after kidney transplantation on graft-related outcomes.

**Methods:**

Our retrospective analysis included all patients receiving their transplant (2007-2017) and receiving their long-term follow up at our center. Clinic nonattendance was extracted from electronic patient records and informatics systems, with highest clinic nonattenders stratified at the 75th percentile.

**Results:**

Data were analyzed for 916 kidney allograft recipients, with median follow up 1168 days (interquartile range, 455-2073 days). Median number of missed transplant clinic visits in the first year was 5 (interquartile range, 3-7) and nonattenders were defined above the 75^th^ percentile. Nonattenders versus attenders were more likely to be black, ABO-incompatible, repeat kidney transplant recipients but less likely to have pretransplantation diabetes. Nonattenders versus attenders had longer hospital stays after their transplant surgery in days (14.4 vs 12.2 respectively, *P* = 0.007), higher rate of delayed graft function (21.3% vs 12.8% respectively, *P* = 0.005), higher risk for 1-year rejection (12.5% vs 7.8% respectively, *P* = 0.044), worse 1-year estimated glomerular filtration rate in mL/min (47.0 vs 54.1, respectively, *P* = 0.002) and increased risk for death-censored graft loss by median follow (17.5% vs 12.0%, respectively, *P* = 0.013). In a Cox regression model, kidney transplant recipients defined as clinic nonattenders within the first postoperative year demonstrated a significantly increased rate of death-censored graft loss (hazard ratio, 1.983; 95% confidence interval, 1.061-3.707; *P* = 0.032).

**Conclusions:**

Kidney transplant recipients in the top quartile for nonattendance require additional support and supervision to help attenuate long-term risks to their graft function and survival.

Nonadherence to immunosuppression after kidney transplantation is common, with rates between 28% and 52% reported in a systematic review of 38 articles,^1^ and a meta analysis of cohort studies has shown a sevenfold increase in risk for graft loss in nonadherent versus adherent kidney transplant recipients.^2^ However, nonadherence after kidney transplantation can also reflect nonattendance to outpatient clinics, which is an essential part of long-term care but a significant investment of time and energy for patients. Missed hospital appointments is a major issue for healthcare providers. For example, in the year 2016 to 2017 there were approximately 8 million missed National Health Service (NHS) appointments in the United Kingdom at a cost of £1 billion (£120 per missed appointment), which is a significant drain on healthcare resources.^3^ The Health and Social Care Information Center in England recently published data about repeated missed appointments, observing 1 in 50 patients who missed an appointment failed to attend 3 or more further appointments within 3 months.^4^

Although data linking missed hospital clinic appointments and adverse outcomes is well established in the medical, surgical and psychiatric literature, there is a paucity of data in the context of solid-organ transplantation. Goodall and colleagues^5^ analyzed nonclinic attendance as part of their analysis into intrapatient variability of tacrolimus levels among 628 kidney transplant recipients in a single-center retrospective analysis of prospectively collected data. They observed the median number of clinic nonattendance comparing patients with high versus low intrapatient variability was 4 (range, 0-22) versus 2 (range, 0-17) respectively (*P* < 0.001). In a multivariate analysis of factors associated with allograft failure, clinic nonattendance was found to be an independent factor (odds ratio, 1.1013; 95% confidence interval [CI], 1.0097-1.2014, *P* = 0.0295). No other published reports have analyzed clinic nonattendance after kidney transplantation, as the focus of clinical research in the area of nonadherence has been exclusively on medication adherence.

We need to understand whether clinic nonattendance is associated with inferior graft outcomes after kidney transplantation and we believe there is a paucity in the transplant literature with regard to this. This is important because identifying clinic nonattendance could be a simple measure of nonadherence, allowing targeted resource allocation to support such kidney transplant recipients. Therefore, the aim of this study was to analyze the impact of clinic nonattendance on posttransplantation outcomes in a large single-center analysis to gain a better understanding of the incidence, risk factors and outcomes for clinic nonattendance within the first year after kidney transplantation.

## MATERIALS AND METHODS

### Study Population

We undertook a retrospective analysis of all consecutive kidney-alone transplants performed at a single transplant center between January 2007 and January 2017. Survival analysis was censored to event or September 2017 (whichever occurred first). We excluded multiple organ transplant recipients and our cohort only included kidney-alone allograft recipients aged 18 years and older with follow up care not repatriated to referring hospitals; all other kidney allograft recipients were included for analysis. Data were electronically extracted by the Department of Health Informatics for every study recruit, with manual data linkage to additional electronic patient records to obtain a more comprehensive data set. Patient and graft survival outcomes, delayed graft function rates and rejection data were acquired and linked with data from the UK Transplant Registry held by NHS Blood & Transplant.

### Definition of Clinic Nonattendance

Data on clinic nonattendance were extracted from the electronic patient record system and was recorded for patients with a scheduled clinic visit that they did not attend (and failed to notify their nonattendance in advance). Nonattendance episodes due to a current inpatient stay were excluded (although these data were only obtainable for inpatient stays at the transplant hospital). We defined the median (± interquartile range [IQR]) number of missed transplant clinic visits within the first year and kidney transplant recipients were classified as nonattenders if their nonattendance rates were above the 75^th^ percentile within the first year posttransplant.

Clinic visits schedules were in line with published guidance from the Renal Association; 2-3 times weekly for the first month, 1 to 2 times weekly for months 2 to 3, every 2 to 4 weeks for months 4 to 6, every 4 to 6 weeks for months 6 to 12 and 3 to 6 monthly thereafter.^6^

### Immunosuppression Protocol

All patients received the same immunosuppression with minimization of tacrolimus exposure, in line with the SYMPHONY protocol^7^ over the study period. Induction therapy was with basiliximab (20 mg ×2) and methylprednisolone (500 mg). Maintenance therapy started from day 0 (operation day) included tacrolimus (target 12-hour trough level 5-8 ng/L), mycophenolate mofetil (MMF, 2 g daily with tapering to 1 g daily after 6-months) and maintenance corticosteroids. Biopsies were indication-based in the context of transplant dysfunction (categorized as ≥20% creatinine rise or new-onset proteinuria). Biopsy data were classified in accordance to latest Banff criteria.^8^ Episodes of acute cellular rejection were treated with a bolus of corticosteroids, with T cell depletion therapy for steroid-resistant rejection. Antibody-mediated rejection was treated with antibody removal by plasmapheresis ± IVIG. Viral serology (eg, polyoma virus) and/or anti-HLA antibodies were checked by indication-basis based upon transplant dysfunction.

### Definitions of Variables

Baseline and posttransplant data were extracted and classified from our database as follows. HLA mismatch levels were defined and graded in accordance to NHS Blood and Transplant classification (level 1: 000, level 2: 100, 010, 110, 200, 210, level 3: 020, 120, 220, 001, 101, 201, 011, 111, 211, and level 4: 021, 121, 221, 002, 102, 202, 012, 112, 212, 022, 122, 222). Matchability is calculated from a standardized pool of 10 000 recent donors, from which the number of blood group identical donors that recipients are well or favorably HLA-mismatched are counted. This number is converted to a standardized score between 1 and 10, which is used to categorize recipients into 1 of 3 matchability groups; easy (1-3), moderate (4-6) or difficult (7-10) to match. Determination of socio-economic deprivation was based upon the Index of Multiple Deprivation, a multiple deprivation model calculated at the local level area. The model is a composite construct of multiple domains reflective of area socio-economic deprivation including (1) income deprivation, (2) employment deprivation, (3) health deprivation and disability, (4) education skills and training deprivation, (5) barriers to housing and services, (6) living environment deprivation, and (7) crime. Individual domains are measured in isolation and subsequently combined (utilizing appropriately judged weighting) into a single composite termed the Index of Multiple Deprivation. On the Index of Multiple Deprivation quintile scale, 1 represented the most deprived and 5 the least deprived area respectively.

### Statistical Analysis

Univariate comparisons done with χ^2^ tests for categorical data, *t* tests or 1-way ANOVA for parametric continuous data, and Wilcoxon or Krustwal-Wallis tests for nonparametric continuous data. All-cause graft failure was taken as the time from transplantation to graft nephrectomy or return to dialysis, whichever was earlier, or death of the patient with a functioning graft. Survival of the patient was defined as the time from transplantation until death. Follow-up analysis of the entire transplant study cohort included all data up to September 2017. With the assumption that any missing data was random, we performed list-wise deletion and excluded the missing values from the analysis.

Log cumulative hazard plots showed no evidence of nonproportionality of hazards. Kaplan-Meier curves were used to show patient and graft survival. All tests were 2-sided and *P* values of less than 0.05 were judged to be significant. Cox proportional hazards regression models were fitted by a stepwise variable selection method to analyze the combined effect of factors on graft survival, reported as hazard ratios (HR). We stratified all kidney transplant recipients by nonattendance by splitting patients into the highest quartile versus all others. Variables of interest that were not found to have significant effects were added individually to the final model. Baseline variables included; age, gender, ethnicity, HLA level, socio-economic deprivation, diabetes as cause of kidney failure, type of donor (living vs deceased), repeat transplant, ABO-incompatibility, nonattendance. Events within the first year added sequentially included clinic nonattendance, delayed graft function, 1-year rejection and 1-year graft function (defined as estimated glomerular filtration rate in mL/min).

### Approvals

This study received institutional approval and was registered as an audit (audit identifier; CARMS-12578). The corresponding author had full access to all data.

## RESULTS

Data were extracted by the Department of Health Informatics for 1421 consecutive kidney allograft recipients between January 2007 and January 2017. Excluding patients who were repatriated back to referring hospitals, we were left with a cohort of 916 kidney allograft recipients for analysis. Median follow-up for this cohort was 1168 days (IQR, 455-2073 days). The median number of missed transplant clinic visits in the first year was 5 (IQR, 3-7); nonattenders were subsequently classified as the high-risk group for clinic nonattendance if their rates were above the 75th percentile (n = 230) with the remainder classed as attenders (n = 686). There was no difference in timing of missed clinic attendance between nonattenders versus attenders; 21.3% and 24.9% of missed clinics were within the first 3-months for nonattenders versus attenders respectively (*P* = 0.602).

### Baseline Comparison

Table 1 compares baseline variables for kidney transplant recipients defined as clinic attenders versus those defined as clinic nonattenders. Clinic nonattenders were statistically more likely to be ABO-incompatible, repeat kidney transplant recipients but less likely to have diabetes at transplantation. From an ethnicity perspective, more black recipients were classified as nonattenders compared to nonblack recipients (36.5% vs 19.6% respectively, *P* = 0.002). Clinic nonattenders also resided closer to the transplant center compared to clinic attenders. No other significant difference was observed in either donor-, recipient- or transplant-related variables.

**TABLE 1:**
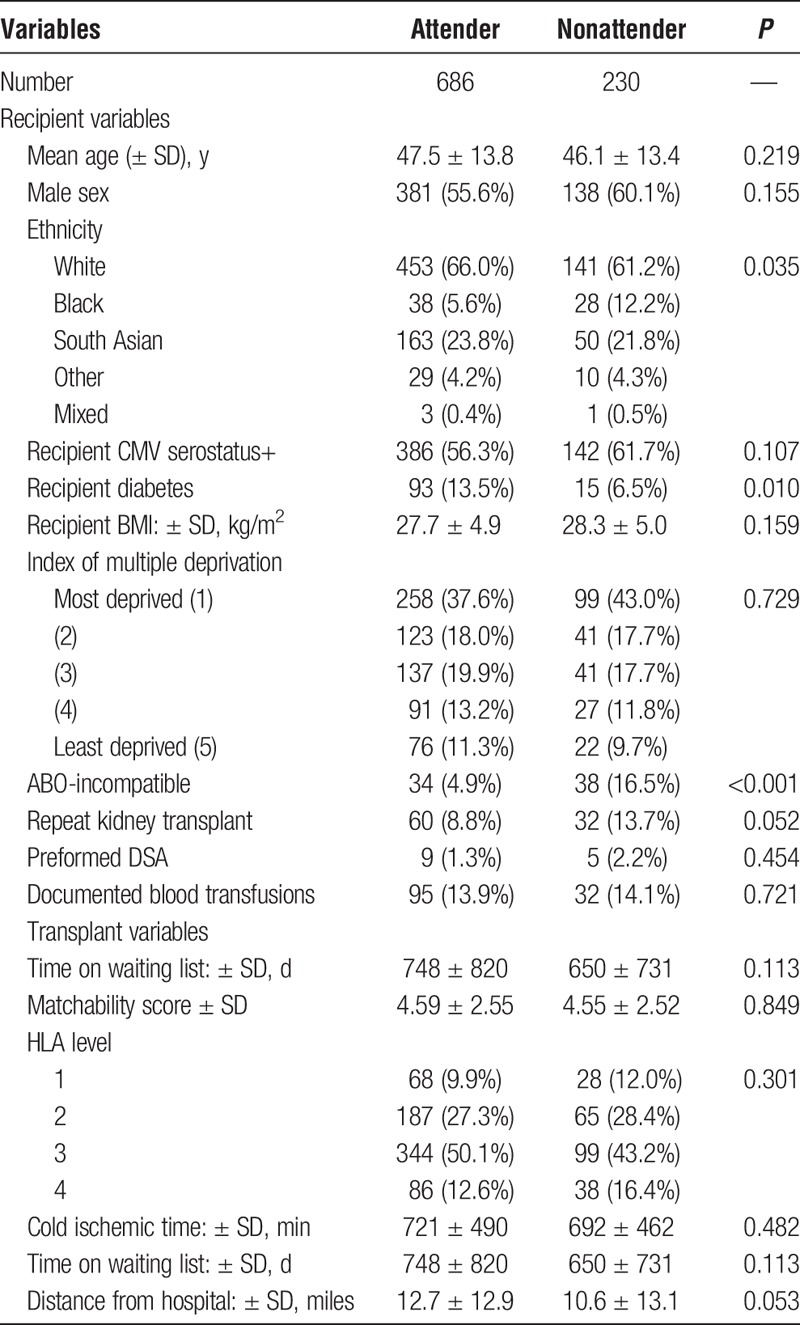
Baseline demographics stratified by nonattendance rates

### Perioperative and Postoperative Events

Table 2 highlights some of the posttransplant related outcomes comparing clinic attenders versus nonattenders and provides evidence that kidney transplant recipients who were subsequently classified as clinic nonattenders were more likely to have traumatic immediate postoperative course. Clinic nonattenders versus attenders had longer hospital length of stay in days during their transplant surgery (14.4 vs 12.2 respectively, *P* = 0.007), possibly driven by a higher rate of delayed graft function (21.3% vs 12.8% respectively, *P* = 0.005).

**TABLE 2:**
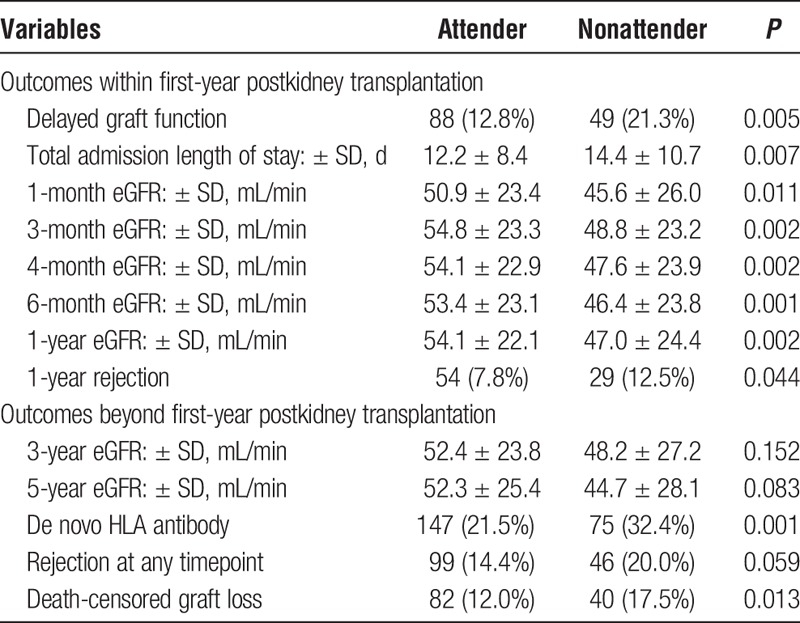
Posttransplant outcomes stratified by nonattendance rates

### Graft-related Outcomes

We observed a higher risk for 1-year rejection among clinic nonattenders versus attenders (12.5% vs 7.8% respectively, *P* = 0.044) and increased risk for rejection at any time point after kidney transplantation (20.0% vs 14.4% respectively, *P* = 0.059). Clinic nonattenders tended to have worse graft function at any time point between 1 month and 5 years (see Table 2).

Donor-specific antibody was checked in 72.3% of kidney transplant recipients at any point after kidney transplantation (checked in the context of allograft dysfunction). Clinic nonattenders were more likely to have a positive donor-specific antibody result compared to clinic attenders at any time postkidney transplantation (32.4% vs 21.5% respectively, *P* = 0.001).

**FIGURE 1:**
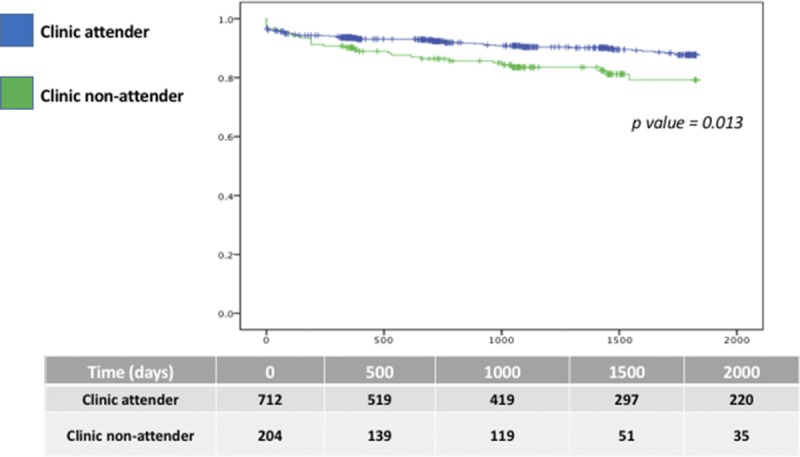
Unadjusted Kaplan-Meir plot of death-censored graft loss comparing clinic attenders versus nonattenders postkidney transplantation.

In our unadjusted Kaplan-Meier analysis, we observed an increased risk for death-censored graft loss by median follow after kidney transplantation among clinic nonattenders versus attenders (17.5% vs 12.0% respectively, *P* = 0.013) (see Figure 1).

Analyzing clinic nonattendance as a continuous variable, we noted increased mean non attendance clinic visits for patients with versus without delayed graft function (7.6 vs 6.3, respectively, *P* = 0.004), risk for 1-year rejection (7.6 vs 6.5, respectively, *P* = 0.044) and risk for death-censored graft loss (7.2 vs 6.4 respectively, *P* = 0.094).

### Multivariate Analysis

We undertook a multivariate analysis using Cox regression models to explore the impact of clinic nonattendance, adjusted for other variables, on death-censored graft loss. We created a series of Cox regression models, starting with baseline variables and individually adding first-year posttransplant events including clinic nonattendance (see Table 3). When just baseline (pretransplant) variables are analyzed, only the following variables were found to be significant for graft loss; cold ischemic time, recipient age and ABO-incompatible kidney transplantation.

**TABLE 3:**
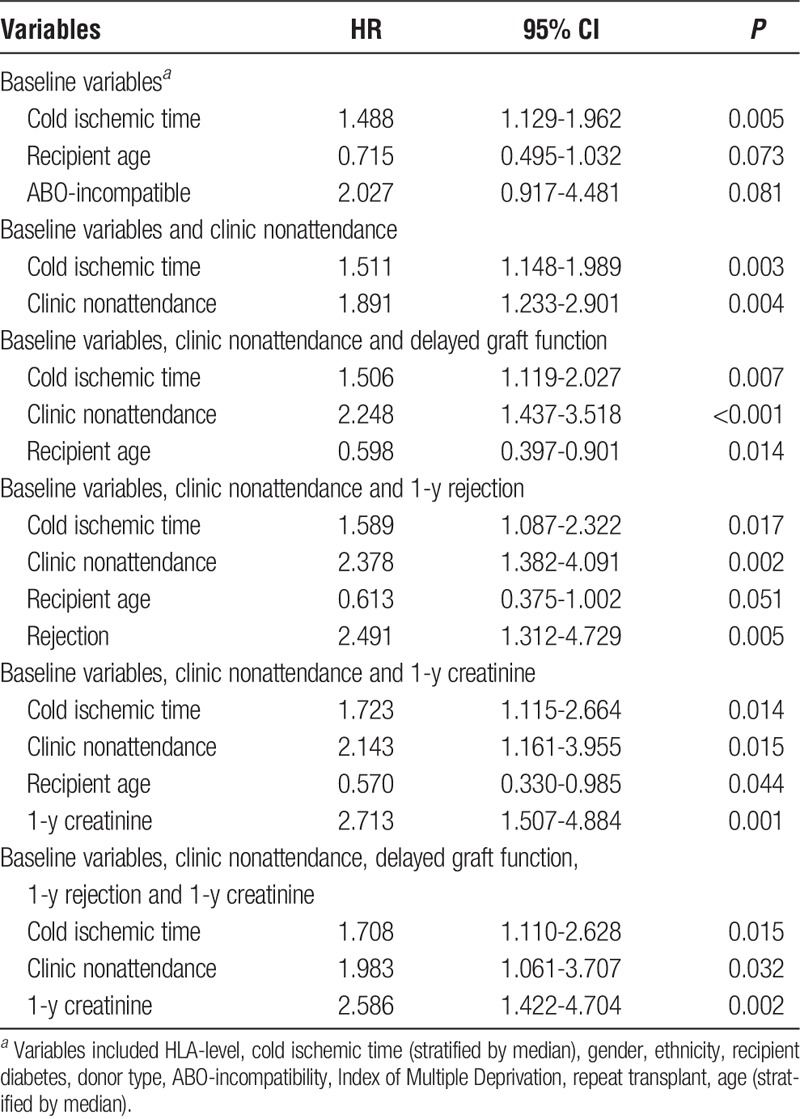
Results of Cox regression models of death-censored graft loss with baseline variables and first year graft-related events

Entering clinic nonattendance as a dichotomous variable (stratified by highest quartile of clinic nonattendance being deemed nonattender), we found clinic nonattendance within the first year posttransplant was independently associated with increased risk for graft loss (HR, 1.891; 95% CI, 1.233-2.901; *P* = 0.004). As clinic nonattendance had an association with posttransplant events such as delayed graft function, 1-year rejection and 1-year graft function (which themselves are associated with graft loss), we included these in subsequent Cox regression models and found clinic nonattendance remained significant in every model (including all variables together; hazard ratio, 1.983; 95% CI, 1.061-3.707; *P* = 0.032).

## DISCUSSION

In this retrospective single-center study, we have explored the risks associated with frequent nonattendance to transplant clinic after kidney transplantation and subsequent posttransplant outcomes. Kidney transplant recipients identified as nonattenders (highest quartile for missed clinic attendance within the first year posttransplant) were more likely to be black, nondiabetic, ABO-incompatibility, repeat kidney transplant recipients and tended to live closer to the transplant center. Nonattenders had more difficult postoperative periods with increased risk for delayed graft function and length of hospital stay. Graft function was poorer at every time point over the subsequent 5-year posttransplant period and clinic nonattenders were more likely to develop anti-HLA antibodies, rejection or graft loss. In a Cox regression model, kidney transplant recipients defined as clinic nonattenders within the first postoperative year demonstrated a significantly increased rate of death-censored graft loss. Our data suggest clinic nonattenders are a high-risk group for adverse graft outcomes after kidney transplantation, warranting close supervision and additional support to attenuate long-term risks to their kidney allograft.

The transplant literature is surprisingly limited with regard to data concerning long-term outcomes for patients with poor attendance to transplant clinic. Goodall and colleagues^5^ have reported similar data to our findings, with clinic nonattendance post kidney transplantation associated with adverse graft-related outcomes including developing donor-specific antibody, rejection and/or graft loss. In a multivariate analysis of factors associated with allograft failure, clinic nonattendance was found to be an independent factor similar to our analyses (odds ratio, 1.1013; 95% CI, 1.0097-1.2014; *P* = 0.0295). Clinic nonattendance was extracted from an integrated, computerized, hospital information system and the mean number of clinic nonattendance was 3.79 ± 3.61 (median 3) between November 2005 and December 2012. Apart from an association between clinic nonattendance and high intrapatient tacrolimus variability, there was no further data to help identify potential clinic nonattenders. However, this study is important for linking nonadherence to immunosuppression with nonattendance to transplant clinic, which appear interlinked with each other. It is therefore very likely that clinic nonattendance is part of the noncompliance spectrum and a surrogate for behavior that should concern transplant professionals. Mindful of the paucity in the transplant literature, our manuscript provides additional insight into the characteristic profile of clinic nonattenders and the subsequent kidney allograft risks associated with nonattendance to transplant clinic.

The link between clinic nonattendance and adverse outcomes has been well reported in the literature, in both primary and secondary care (medical/surgical) settings. At a primary care level, both patient (eg, certain age groups, socio-economic deprivation) and practice (urban practices with 2-3 day waiting times) characteristics have been linked with clinic nonattendance.^9^ Williamson and colleagues^10^ are currently undertaking a proof-of-concept study to try and better understand nonattendance in a primary care setting in an effort to achieve better patient engagement to help reduce missed appointments. Nonattendance has also been frequently described in a variety of hospital outpatient secondary care visits, including medical, surgical, and psychiatric settings, with heterogeneous reasons described by patients for their missed hospital clinic visit.^11^ Nonattendance at secondary care hospital clinics is also poorly communicated to primary care doctors, which could be one simple area of practice improvement to develop more collaborative approaches to improve non-attendance rates.^12^

So what can be done to improve clinic attendance after kidney transplantation? Clearly the necessary intervention will be dependent upon the reasons for nonattendance; for example, nonattendance due to apathy will requires difference resources from nonattendance due to work commitments. Numerous strategies have been proposed across the healthcare sector, with the evidence-base limited for a gold-standard intervention. Reminder interventions (such as text messages, telephone calls, emails, etc.) have been shown to moderately reduce clinic nonattendance in a systematic review of largely observational studies.^13^ Flexible booking mechanisms, such as the choose-and-book system utilized in the United Kingdom by primary care doctors booking hospital outpatient appoints appears to have reduced clinic nonattendance rates by 8.7%.^14^ Ellis and colleagues^15^ found hospital outpatient clinic nonattendance rates are highest on a Monday with sequential decrease over the course of the week (with lowest nonattendance rates on Friday), suggesting preferential allocation of appointment days may be of benefit to reduce clinic nonattendance. Offering financial incentives for clinic attendance would be an expensive intervention but lacks clear evidence; in fact it may achieve the opposite effect. For example, Judah and colleagues^16^ found clinic nonattendance for diabetic eye screening was actually worse in the group offered financial incentives. Even reciprocal financial strategies, such as punitive measures, lacks evidence-base. Blaehr and colleagues observed no demonstrable difference in clinic nonattendance in a randomized controlled trial conducted at a regional hospital in Denmark, where users were either subject to nonattendance fines (with advance notification with appointment letters) or standard of care for their clinic attendance.^17^

Perhaps the most encouraging intervention may be related to cognitive behavior intervention to support self-management. Jamieson and colleagues^18^ have conducted a systematic review of the literature to explore motivations and attitudes of kidney transplant recipients to self-management. Five themes were identified in their thematic synthesis of published research: (1) empowerment through autonomy, (2) prevailing fear of consequences, (3) burdensome treatment and responsibilities, (4) overmedicalizing life, and (5) social accountability and motivation. From these findings, it is clear that multicomponent interventions incorporating personalized care planning, education, psychosocial support, decision aids, and self-monitoring tools will be required to foster self-management capacity with the aim of improving long-term outcomes after transplantation. Such self-management techniques are important as Massey and colleagues, in the context of medication nonadherence, have identified discrepancies between adherence beliefs and actual behavior among kidney transplant recipients in qualitative studies.^19^ Self-management interventions in the context of chronic illness conditions should be an important aspect of care for kidney transplant recipients, with numerous methodological techniques available,^20^ and should be facilitated by clinical teams to improve adherence and subsequent outcomes. However, further research is warranted to determine whether such cognitive behavior interventions actually can lead to a significant difference in clinic nonattendance after kidney transplantation.

The limitations of our study should be appreciated in the interpretation of our data. Nonattendance rates were determined from electronic patient records and clerical errors may have falsely defined some nonattendance (eg, double-booked clinics, scheduled clash with another appointment, etc.), with no underlying etiology available to explain reason for nonattendance. Understanding actual reasons for failed clinic visits will be important for any future work in this area. While inpatient episodes were checked to adjust for any clinic nonattendance visits, this was not possible for inpatient episodes at other hospitals due to the lack of IT linkage to other hospital electronic patient records. We did not account for missed clinics other than within the transplant service and this will confound the data, especially for patients with multiple comorbidities and significantly more hospital outpatient visits. Evidence of pretransplant noncompliance would be important to ascertain as it likely is a major confounder on the risk for clinic nonattendance. Unfortunately, clinic nonattendance data are not useful for the large cohort of recipients on hemodialysis pretransplant as clinical reviews are done during dialysis sessions and alternative definitions would introduce subjective bias. Typical of epidemiological analyses of cohort studies such as these, there are likely to be confounders that have an impact on graft function and loss postkidney transplantation that we were unable to factor in (eg, lifestyle factors, travel costs, etc.). Missing data (and misclassification bias) also has an implication on the analyses performed, which is an inherent bias in epidemiological analyses such as this. Our study was also likely to be under-powered, and of short duration, to robustly assess difference in some outcomes. Given the few graft losses between the 2 strata, multivariable analysis of retrospective data may not capture the full spectrum of risk factors, as highlighted by the large 95% confidence intervals, and further maturing of this database in the long-term may provide more definitive answers in the future.

To conclude, our study has demonstrated clinic nonattendance in the first year after kidney transplantation is a risk factor for adverse graft-related outcomes and identified certain characteristics associated with patients more likely to miss transplant outpatient visits. Kidney transplant recipients with frequent clinic nonattendance in the first year should be considered at high-risk for poor graft-related outcomes, requiring detailed investigation of underlying causes to provide targeted counseling and support as deemed appropriate. However, the ideal intervention to reduce clinic nonattendance has not been elucidated and requires further clinical research. We believe transplant-specific interventions aimed at reducing transplant clinic nonattendance rates should be developed, which may subsequently have a positive impact at attenuating adverse graft-related outcomes in the long-term, but requires deeper understanding of the underlying causes for missed clinic attendance to target interventions effectively.
